# Cosmic radiation shielding property of boron reinforced continuous fiber nanocomposites produced by electrospinning

**DOI:** 10.1186/s11671-023-03940-3

**Published:** 2023-12-11

**Authors:** Mücahid Özcan, Cengiz Kaya, Figen Kaya

**Affiliations:** 1 Mechanical Engineering Department, Faculty of Engineering, Adıyaman University, Adıyaman, Turkey; 2 Department of Metallurgical and Materials Engineering, Faculty of Chemistry and Metallurgy, Yıldız Technical University, 34210 Istanbul, Turkey

**Keywords:** Radiation shielding, Nanocomposite, Electrospinning, Polyvinyl alcohol, Boron

## Abstract

Electrospinning, a cutting-edge production technique, is used to create boron-reinforced continuous fiber nanocomposites that shield space missions from cosmic radiation, a significant hazard. By incorporating boron, which is known for its exceptional neutron shielding properties, into the polymer matrix, a composite material that is flexible, lightweight, and highly resistant to radiation is produced. The results indicate that continuous fiber nanocomposites reinforced with boron, boric acid, or both have a high shielding efficiency against cosmic radiation. The adaptability and low weight of the manufactured nanocomposites make them ideal for space applications. While boric acid combines with PVA at the molecular level and alters the molecular chain structure of PVA, it is believed that elemental boron is only incorporated as particulates into the PVA polymer. It is known that both boric acid and elemental boron doped nanocomposites provide samples with a thickness of 10 microns with 13.56% neutron shielding and superior photon blocking ability.

## Introductıon

In recent years, humanity’s fascination with space has increased significantly. Nevertheless, the development of radiation-shielding wearable technologies is crucial [1, 2]. Moreover, “wearable technologies” refers to devices that shield users from radiation, including X-rays, gamma rays, and neutron particles. Demand for radiation-shielding materials is anticipated to peak during the Mars colonization process and subsequent periods [3, 4]. Electronic equipment is now a component of the cutting-edge equipment astronauts wear during spacewalks. Ionizing particles are capable of interfering with the functionality of advanced technological devices and pose significant threats to human health. Consequently, cosmic radiation is of the uttermost importance in the design of space-wearable technologies [3, 5].

According to the Bethe-Bloch formula, materials with a high ratio of atomic number to atomic mass are more effective at absorbing radiation [6]. Radiation is divided into primary and secondary categories according to its capacity to initiate and combine multiple types of radiation. Radiation that travels directly from the source to the shielding material is referred to as primary radiation. In contrast, secondary radiation is produced when primary radiation interacts with the elements of the shielding material. Due to the large difference in energy levels between secondary and primary radiation, shielding materials must contain elements with high atomic numbers [7, 8, 9, 10].

Due to its high ratio of atomic number to atomic mass, hydrogen is regarded as the most effective and fundamental component for radiation shielding. In order to fabricate polymer nanocomposites utilized for radiation shielding [11, 12, 13], the use of polymers with a high level of hydrogen becomes essential. In terms of shielding cosmic radiation, polyvinyl alcohol has numerous advantages, including its formability, high hydrogen content, and cost-effectiveness. In addition to ionizing radiation such as X-rays and gamma rays, non-ionizing neutron particles are widely regarded as the most dangerous type of radiation due to their potential to cause severe damage to human health [14–18]. Lithium, cadmium, and boron are advantageous neutron shielding materials because they have a large neutron absorption cross-sectional area. Boron is a suitable choice despite having a relatively smaller absorption cross-sectional area than other elements [14, 19–21] due to its low cost of production and ease of application.

Boron's incorporation into continuous fiber nanocomposites demonstrates the need to go beyond conventional radiation shielding techniques [22, 23]. Utilizing boron's superior properties, these novel techniques substantially improve the material's radiation attenuation efficiency [24–26]. It takes advantage of the distinctive properties of boron, which are renowned for their exceptional neutron absorption capacities. Importantly, the electrospinning technique guarantees the uniform distribution of boron in the polymer matrix, while the ability to precisely manipulate the nanostructure morphology permits the formation of dense and nonwoven fiber mats [14, 24, 27].

Recent studies have shown that composites with fiber morphologies characterized by low density, high impact resistance, and tensile strength have superior shielding capabilities than conventional bulk materials. Therefore, nanofibers are produced by electrospinning, which is considered the simplest and most cost-effective method for fiber production [28, 29]. In our previous research, the addition of boric acid to polyvinyl alcohol (PVA) solution improved the shielding performance of PVA nanofibers reinforced with boron. It has been established that the neutron shielding efficacy of 10-micron-thick borate ester PVA nanofibers produced with boric acid as an additive is at least 6% [24]. The importance of achieving uniform fiber formation in shielding was emphasized, along with the observation that the percentage of shielding displayed a positive correlation with the high boron content in the molecular composition of the fibers. Consequently, the use of uniform fibers containing a significant quantity of boron is anticipated to increase the effectiveness of shielding [14].

In this study, the combined use of boric acid and elemental boron in polyvinyl alcohol (PVA) nanofiber compositions, as well as the synergistic effect of these two materials on the nanofiber structure and radiation protection properties, are thoroughly investigated. Changes in viscosity and fiber diameter, as well as structural changes resulting from morphological changes caused by varying boric acid and elemental boron concentrations, are analyzed in detail. In wearable technology applications, ensuring the optimal elemental boron concentration to enhance the radiation protection properties of nanofibers by preserving their fiber structure provides an effective equilibrium between structural integrity and radiation protection efficiency. The molecular and morphological changes brought about by nanocomposites produced from compositions containing both boric acid and boron demonstrate that the shielding efficiency exceeds 13%, highlighting the applicability of the developed nanofiber mats in radiation protection applications.

## Materials and methods

### Materials

Polyvinyl Alcohol (Mw = 85,000–124,000,99 + % hydrolyzed), Boric acid for analysis (ACS, ISO, Reag. Ph Eur), and Ethanol absolute for analysis (ACS, ISO, Reag. Ph Eur) were purchased from Sigma Aldrich and used as received without purification. PVZ Nano Boron (Elemental Boron Powder, Amorphous) with a particle size range of 50–300 nm was purchased from Pavezyum Advanced Chemicals.

### Preparation of borate ester solution

Two different solutions were produced for the synthesis of borate ester nanofibers. By dissolving boric acid in purified water while agitating at 500 rpm, a combination of PVA solutions containing the boric acid equivalent of 1–1, 5–2–3 wt% in the entire solution was produced. A second solution consisted of purified water and 5% PVA that was mixed for two hours at 80 °C and 300 rpm. Using a dropper, the boric acid solution was introduced to the PVA solution. After that, an hour of magnetic agitation was used to produce the borate ester solution [24].

### Preparation of boron-reinforced PVA solution

Following a method described in our prior work, boron-doped PVA nanofiber was created by dissolving PVA polymer in distilled water as a solvent. In this work, ethanol was used to modify the surface of boron particles to improve their adherence to the PVA nanofiber and their integration into the fiber structure. Thus, the PVA solution was dissolved in distilled water at 80 °C with magnetic stirring for 2 h at 300 rpm before adding 0.5–1–2–4 wt% of surface-modified boron particles. The boron-reinforced PVA solution required 24 h of mixing at room temperature to get a homogenous solution and was then prepared for electrospinning [14].

### Preparation of boron-reinforced borate ester solution

PVA solutions were created by dissolving PVA polymer in 5 weight percent purified water at ambient temperature. After dissolving the polymer completely with magnetic stirring for 2 h, the next phase was to add 1—1.5—2% boric acid by weight successively. This created three unique solutions. The names of the solutions were BA1-PVA, BA1.5-PVA, and BA2-PVA. After the PVA polymer had completely dissolved in purified water in another solution, 1% elemental boron was added, and the mixture was stirred for 24 h at 100 revolutions per minute. An hour was spent combining and ultrasonically blending the two prepared solutions to produce a single homogenous solution. There are three different blending solutions: B1/BA1-PVA, B1/BA1,5-PVA, and B1/BA2. Since the polymer structure may degrade due to moisture and temperature, all solutions were prepared and electrospun the day before the experiment. The contents of the compositions are given in Table 1.

**Table 1:** The description and component content of the compositions

Sample Code	Composition
Neat PVA	5 wt% PVA (in solution)
BA1-PVA	1 wt% boric acid + 5 wt% PVA (in solution)
BA1,5-PVA	1,5 wt% boric acid + 5 wt% PVA (in solution)
BA2-PVA	2 wt% boric acid + 5 wt% PVA (in solution)
B0,5-PVA	0,5 wt% elemental boron + 5 wt% PVA (in solution)
B1-PVA	1 wt% elemental boron + 5 wt% PVA (in solution)
B2-PVA	2 wt% elemental boron + 5 wt% PVA (in solution)
B1/BA1-PVA	1 wt% elemental boron + 1 wt% boric acid + 5 wt% PVA (in solution)
B1/BA1,5-PVA	1 wt% elemental boron + 1,5 wt% boric acid + 5 wt% PVA (in solution)
B1/BA2-PVA	1 wt% elemental boron + 2 wt% boric acid + 5 wt% PVA (in solution)

### Electrospinning procedure

**Fig. 1:**
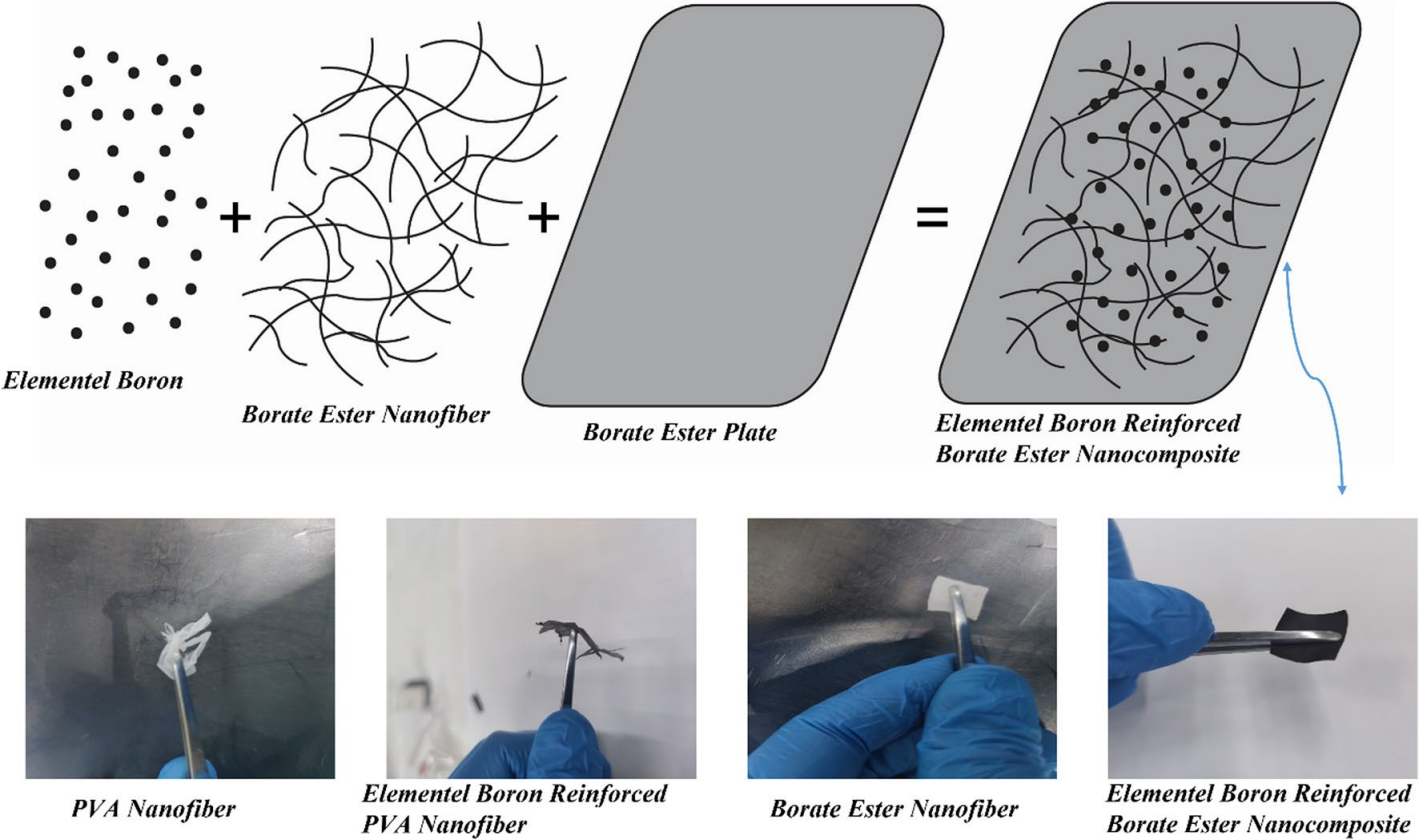
Formation and differentiation of composite structure during electrospinning

In this study, the solution parameters were altered to control the nanofiber morphology, while the electrospinning parameters remained unchanged. The electrospinning system is comprised of a syringe pump with 28 l/min feed rates, an aluminum cylinder drum collector rotating at automatically controlled operation velocities, and 15 kV of applied potential power. 20 gauge electrospinning needles were used to insert solutions into 20 cc plastic syringes using stainless steel needles. The fiber produced at the syringe tip of stainless steel was collected at an aluminum target located 15 cm from the tip. The fiber mats that had accumulated on the Al target were electrospun at room temperature, peeled off, and then desiccated at 70 °C for 24 h (Fig. 1).

### Radiation shielding properties investigations

**Fig. 2:**
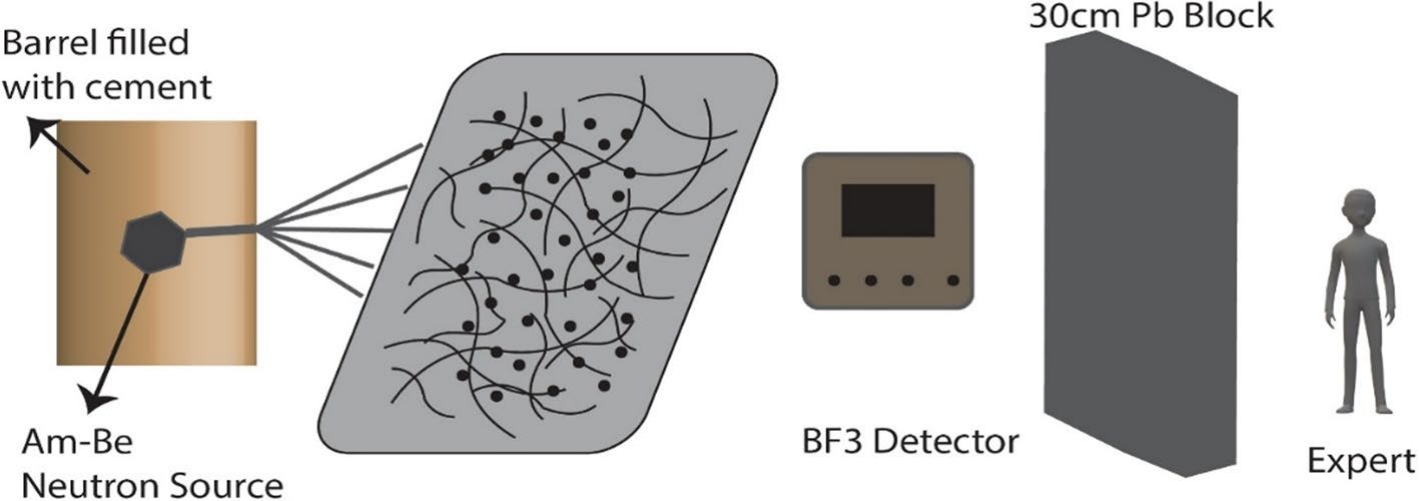
Neutron Shielding Process

An expert team from the Kücükcekmece Nuclear Research Center measured neutron shielding. In a sustainable manner, the (241 Am/9 Be) of the 2 Ci neutron source is stored in the center of a container filled with cement (Fig. 2). The neutron source and sample interact when the cement portion in the source's opened barrel is removed for measurement. Three 120-s measurements were taken with the Thermo Scientific RadEye NL detector, and the average of these measurements was then calculated. Without the sample, neutrons reaching the detector directly from the source were measured, and three independent measurements were taken for each sample to ascertain its shielding capacity. Since the neutron particle poses a grave threat to human health, the specialist crew conducts their duties behind 30 cm-thick lead blocks.

A theoretical investigation of gamma shielding performance was conducted using Phy-X/PSD software [30, 31]. The utilized equations for computations were documented in previously published research. The shielding properties of gamma radiation were determined by utilizing formulation-derived values of key variables, including the linear attenuation coefficient (LAC), mass attenuation coefficient (MAC), and half-value layer (HVL).

1 $$\frac{I}{{I}_{0}}={e}^{-\mu x}$$

I_0_ is the intensity of gamma rays at an absorber thickness of zero, I is the intensity of gamma rays transmitted through an absorber of thickness x, and the linear attenuation coefficient. Based on Eq. 1, the PhyX program has calculated the theoretical values of the mass attenuation coefficients of compounds [32, 33, 34].

2 $${\mu }_{m}=\frac{\mathrm{ln}(\frac{{I}_{0}}{I})}{\mathrm{\rho t}}$$

*μ*_m_ is the mass attenuation coefficient for the compounds, *t* is the thickness of the absorber (cm), and *ρ* is the density of the target (g/cm^3^). Based on Eq. 2, the PhyX program has calculated compounds' theoretical mass attenuation coefficients. [33–35].

3 $${X}_{h}=\frac{ln2}{\mu }$$

The Half Value Layer (HVL) is calculated based on Eq. 3, the PhyX program has calculated the theoretical half-value layer of compounds [32].

### Characterization of polymer solutions and nanofibers

The prepared solutions’ chemical makeup was analyzed using the Fourier transform infrared spectroscopy (FTIR) examination, performed with a Perkin Elmer Spectrum 100 spectrometer. The thermal degradation behavior of nanofibers was made by carrying out thermogravimetric analysis (TGA) that was performed utilizing a Seiko Instruments SII, Exstar 6300 TG/DTA under a nitrogen atmosphere spanning a temperature range of 25–1100 ◦C. The nanoparticles used as reinforcement and produced nanofibers were examined using the Zeiss EVO LS 10 field emission scanning electron microscope (FE-SEM), which was equipped with an energy-dispersive X-ray spectrometer (EDS). Density and specific gravity ASTM D4892 (helium pycnometer method). Standard specifications and operating instructions for glass capillary kinematic viscometers, ASTM D446 (oswald viscometer).

## Results and discussion

### Chemical structure and thermal behavior of PVA nanofiber reinforced with elemental boron and boric acid

**Fig. 3:**
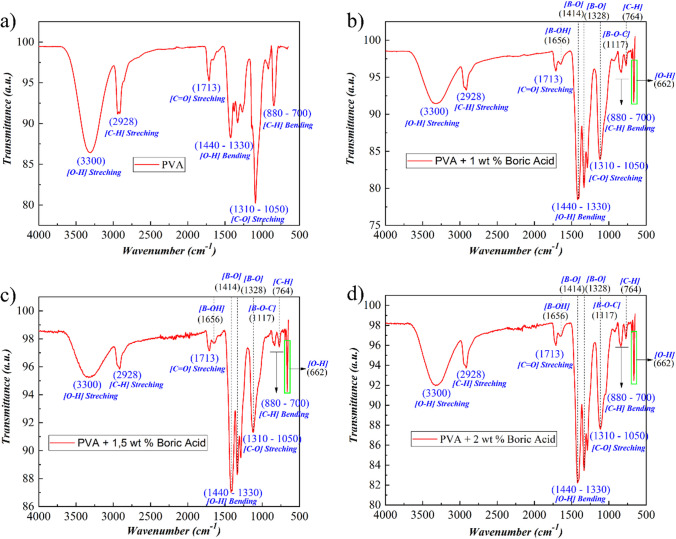
FT-IR spectra of Neat-PVA (**a**), BA1-PVA (**b**), and BA1,5-PVA (**c**), BA2-PVA (**d**), nanofibers

The impact of boric acid addition on the chemical structure and the conversion of PVA nanofibers to borate ester nanofibers were validated by FTIR. As shown in Fig. 3, the addition of boric acid decreased the intensity of the stretching vibration at 3300 cm^−1^, which is related to O–H bonding. The peaks at 2928 cm^−1^ show the C–H bond’s stretching vibration in the PVA structure. In addition, bending vibrations at 1713 cm^−1^ (C=O), 1440–1330 cm^−1^ (O–H), 1310–1050 cm^−1^ (C–O), and 880–700 cm^−1^ (C–H) exhibited characteristic peaks in the PVA molecular structure as determined by FTIR [36, 37]. Boron, which is partially incorporated into the PVA molecule structure by dissolving PVA and boric acid in water, can be detected by FTIR analysis at multiple points, despite the fact that the majority of the characteristic bonds of PVA retain their structure upon addition of boric acid. In accordance with the literature [38–41], when boric acid is added, the bonds at 1656 cm^−1^ (B–OH), 1414 and 1328 cm^−1^ (B–O), and 1117 cm^−1^ (B–O–C) begin to generate prominent peaks in the nanofiber structures. The unambiguous visibility of the (O–H) bond at 662 cm^−1^, one of the characteristic peaks of boric acid, in Fig. 3b to d indicates that boric acid is incorporated into the fiber structure. The addition of more boric acid widened the (O–H) signal at 3300 cm^−1^. Due to the alteration in the molecular chain structure of nanofibers produced by electrospinning a solution containing boric acid and polyvinyl alcohol [24], it is typical for the FTIR spectrum to display a variety of peaks. In addition to the molecular structure interaction, the O–H bond at the 662 cm^−1^ peak is evidence that during electrospinning, a portion of boric acid reverts to the H_3_BO_3_ form.

**Fig. 4:**
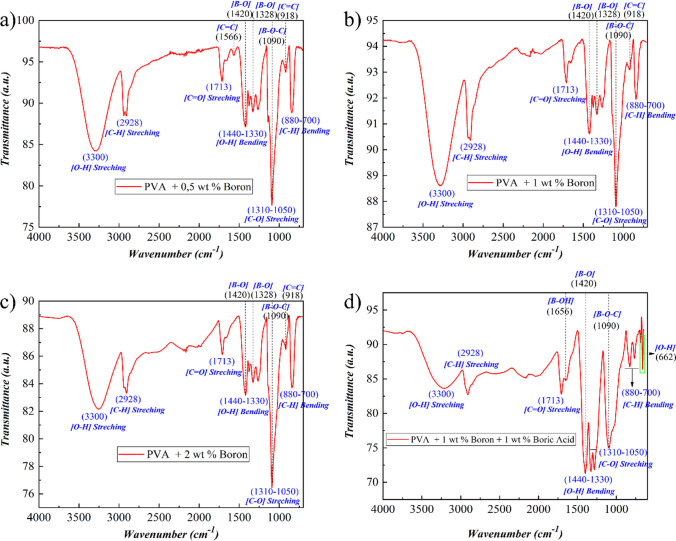
FT-IR spectra of B0,5-PVA (**a**), B1-PVA (**b**), and B2-PVA (**c**), B1/BA1-PVA (**d**), nanofibers

Similar to boric acid addition, boron bonds were also formed at 1420 and 1328 cm^−1^ (B–O) and 1090 cm^−1^ (B–O–C) in PVA nanofibers produced with elemental boron addition [14, 42], as verified by FTIR analysis shown in Fig. 4. The most significant signal of stretching at 3300 cm^−1^ (O–H) in the PVA widened with the addition of elemental boron and boric acid, suggesting that the PVA structure is degrading due to the presence of excess boron in the form of nanoparticles and boric acid molecules. Boron addition to the PVA solution could be surmised to alter the nanofibers' macromolecular structure during electrospinning. Correspondingly, other PVA-structure-related signals, such as (O–H), (C–H), and (C=O) bonds, were also significantly diminished. As shown in Fig. 4, the intensities of FTIR signals related to boron bonds such as (B–O), and (B–O–C) increased.

Both boric acid and elemental boron are acknowledged to interact with PVA. Boron-doped PVA solution does not produce nanofibers with a B–OH bond, indicating that it has a much smaller effect on the molecular structure than boric acid does. While boric acid alters the entire molecular structure of PVA, elemental boron only affects the particle surface, initiating the interaction between the polymer and the metal surface. This not only results in the uniform distribution of elemental boron within the nanofiber, but also increases the composite's strength through strong surface interaction.

### Thermal degradation behavior of PVA nanofiber reinforced with elemental boron and boric acid

**Fig. 5:**
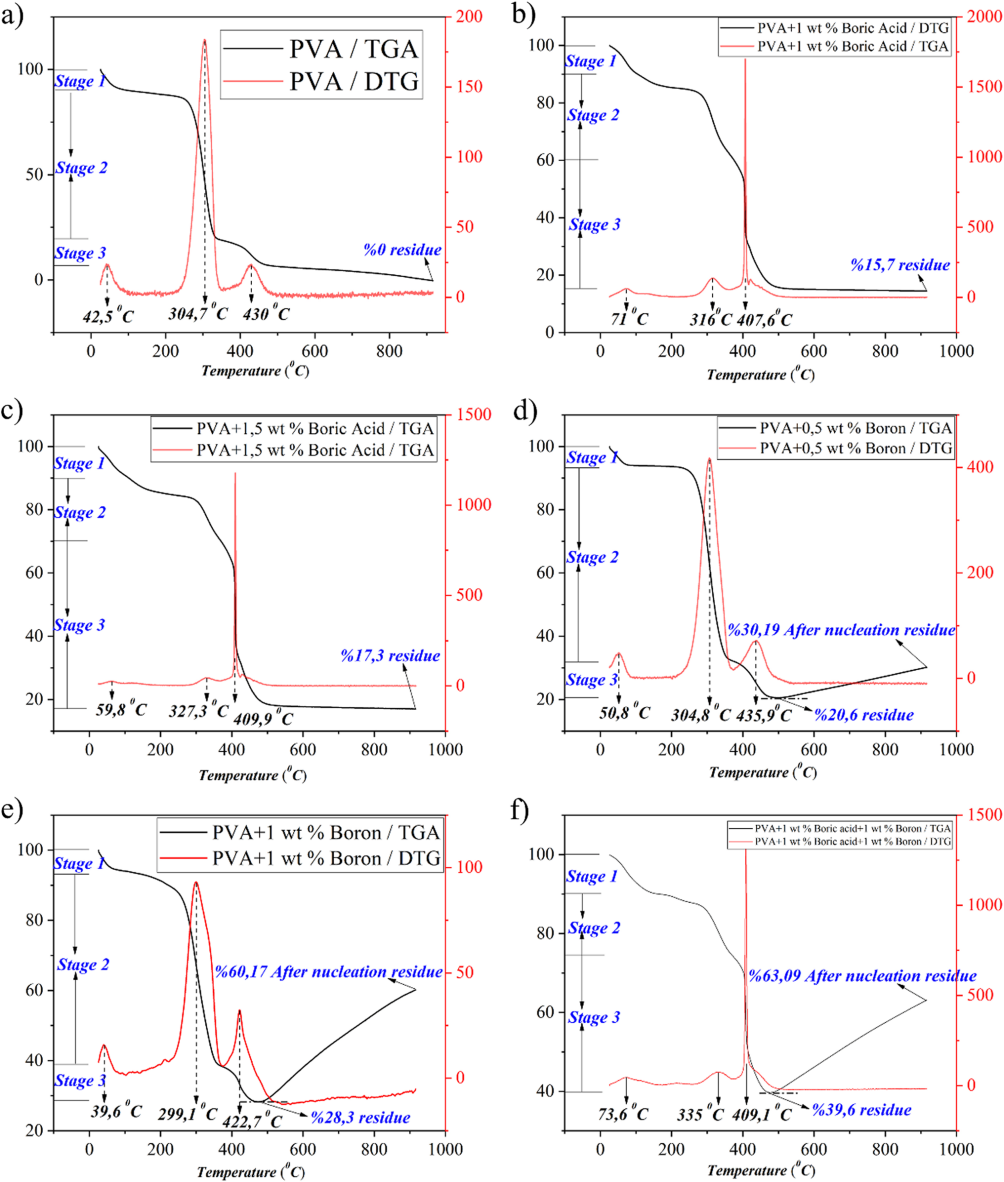
Thermogravimetric analysis results of Neat-PVA (**a**), BA1-PVA (**b**), BA1,5-PVA **c** B0,5-PVA **d** B1-PVA (**e**) and B1/BA1-PVA **f** nanocomposite fibers

Figure 5a demonstrates the four-stage degradation behavior of neat PVA nanofibers. Moisture and solvents are extracted with a 10% mass loss in the first stage. In the second stage, which occurs at an average temperature of 304.7 °C, the side and main polymer chains disintegrate, resulting in a 70% mass loss. In the third and fourth stages [43], the remaining mass completely dissipates as a result of the degredation of the residual organic components. As depicted in Fig. 5b and c, the addition of boric acid increased the high temperature resistance of PVA fibers to over 400 °C, resulting in a 40% total mass loss in the initial and second phases as opposed to the 80% loss in pure PVA. Provides a reduction in heft. Due to the fact that boric acid modifies the molecular structure of PVA and generates a more complex chain structure, the polymer’s resistance to heat increases [44]. After heat treatment at 900 °C, only 16% of the initial mass remains for PVA nanofibers containing 1% boric acid due to reduced thermal degradation. 17% of the initial mass remains after heating PVA nanofibers doped with 1.5% boric acid, as depicted in Fig. 5c, indicating that the thermal resistance improves with increasing quantities of boric acid. Figure 5d, e shows that elemental boron does not affect the molecular chain structure of PVA in nanofibers containing elemental boron and only induces an interaction on the particle surface. Although boron nanoparticle reinforced material experiences a 70% weight reduction at temperatures below 304.7 °C, elemental boron particles oxidize between 500 and 900 °C, producing boron oxide, resulting in a 30% increase in mass. As depicted in Fig. 5e, the quantity of oxidation increases directly in proportion to the amount of elemental boron, resulting in mass increases in excess of 60%. In Fig. 5f, it is seen that in nanofibers containing a mixture of elemental boron and boric acid, 63% mass residue is formed as a result of the side and main chains of the boric acid polymer increasing the thermal resistance by incorporating boron into the molecule structure and the oxidation reactions caused by elemental boron.

### Microstructure of PVA nanofiber reinforced with boron and boric acid

By optimizing electrospinning and solution parameters, the morphology of boric acid and elemental boron-doped PVA nanofibers in terms of fiber diameter, viscosity, and particle-polymer interaction is investigated [45]. Since the addition of boric acid causes extremely high viscosities in the PVA solution, it reduces fluidity and makes electrospinning of nanofibers challenging. Adding elemental boron to PVA solution reduces fibreability by disrupting the chain structures that enable fiber formation. Consequently, it is essential to optimize solution parameters and electrospinning parameters.

**Fig. 6:**
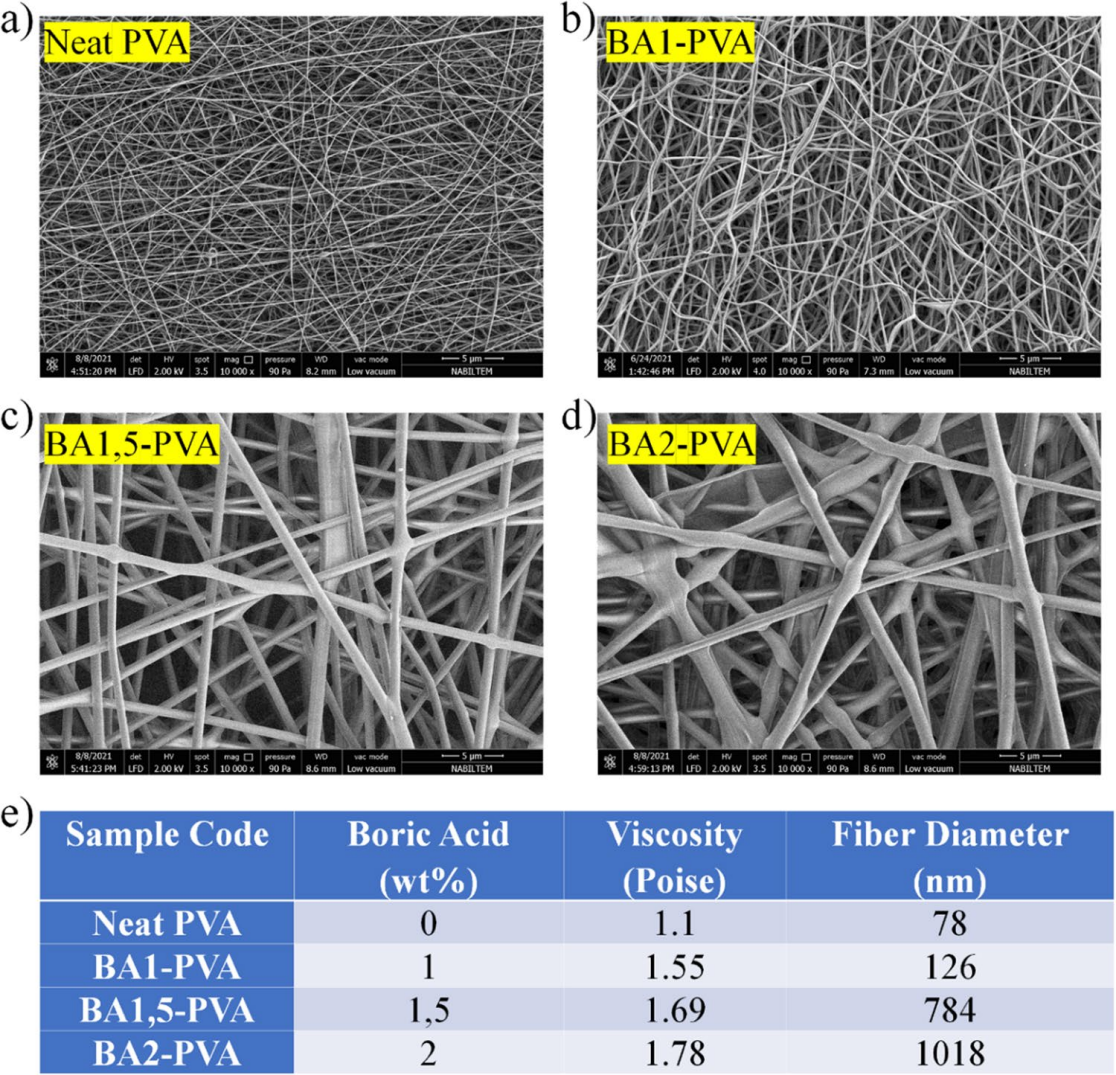
SEM images of boric acid doped PVA nanofibers (Neat PVA (**a**), BA1-PVA (**b**), BA1,5-PVA (**c**), BA2-PVA (**d**)) and viscosity and fiber diameter change according to boric acid addition (**e**)

First, morphology alterations were investigated by adding between 1 and 2% by weight of boric acid to the PVA solution. The addition of boric acid decreased the fluidity of the PVA solution and increased its viscosity. As in prior research [14–21, 24, 28, 29, 42], the researchers discovered that as viscosity increased, fiber diameters progressively increased. The viscosity of the PVA solution increased from 1.15 poise to 1.55 poise when 1% boric acid by weight was added. The diameter of the fibers increased directly in proportion to the viscosity, from 78 to 126 nm. The viscosity attained 1.78 poise after the boric acid additive was gradually increased to 1.5 and 2 weight percent. As shown in Fig. 6, the solution-produced fibers can reach a maximum thickness of 1018 nm when the boric acid concentration in the PVA solution exceeds 2%. It is well known that the increase in fiber thickness caused by electrospinning is directly proportional to a modification of spinning or solution parameters. By progressively increasing the concentration of boric acid and holding all other parameters constant, it is determined that boric acid is the sole factor in fiber thickening. When the quantity of boric acid added to the solution exceeds 2% by weight, the viscosity value exceeds 2.5 poise, exceeding the fiber formation threshold for this solution. For this reason, the maximum contribution amount was selected as 2% by weight.

**Fig. 7:**
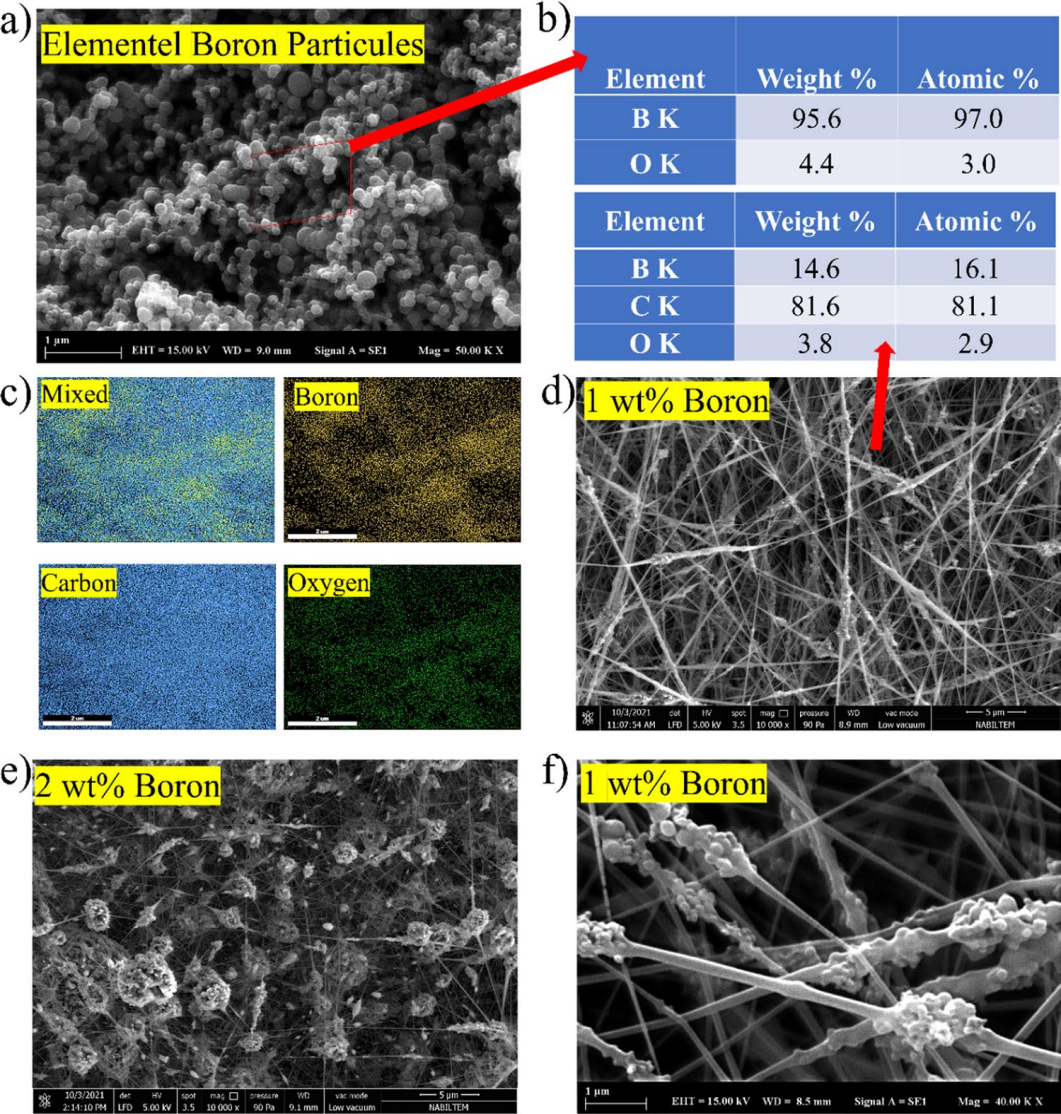
SEM images of elemental boron particles, **a** EDS point of elemental boron particles and EDS point analysis of B1-PVA nanofibers, **b** EDS mapping analysis of B1-PVA nanofibers, **c** SEM images of boron-doped PVA nanofibers (B1-PVA (**d**), B2-PVA(**e**)), HR-SEM images of B1-PVA nanofibers (**f**)

As shown in Fig. 7a and b, the elemental boron used to reinforce PVA nanofibers had a spherical morphology ranging between 50 and 300 nm and a high purity greater than 97 atomic% boron. Boron atoms were chosen for reinforcing PVA fibers because of their spherical structure. At 1 wt% elemental boron addition to PVA nanofibers, the charge applied during electrospinning causes the boron particles to aggregate on the fiber surfaces. As shown in Fig. 7e, these aggregates form large composite spheres when 2 wt% elemental boron is added. The average theoretical composition of composite fibers is 20% elemental boron and 80% PVA fiber structure. As shown in Fig. 7c, EDS analysis of 1 wt% boron reinforced nanofibers revealed that the elemental boron particles were uniformly distributed on the fiber mat, both on the outer surface and within the PVA nanofibers (Fig. 7d). Figure 7f depicts a magnified examination of boron clusters on the PVA nanofibers. As long as certain contribution limits are not exceeded when settling into PVA fiber, it is understood that elemental boron particles are regular and fiber-compatible. Boron particles, which are integrated into almost the complete fiber, were measured to be 14.6% by weight in PVA fiber (Fig. 7e). These EDX ratios indicate that the contribution of boron to the PVA fiber is at its optimum level. In addition, it is known that dispersing boron particles into the fiber will improve the thermal and chemical properties of PVA fiber.

**Fig. 8:**
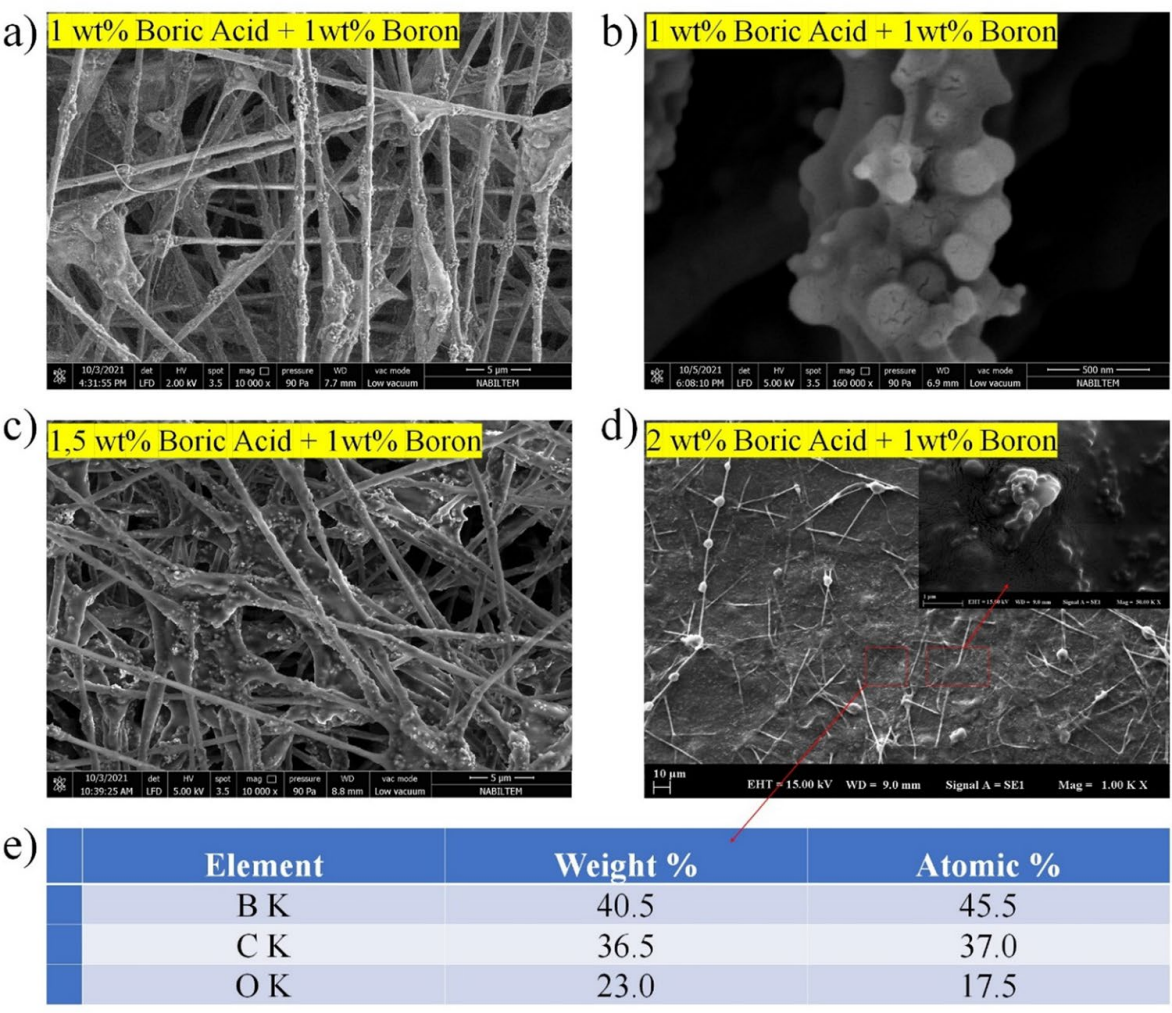
SEM images of boric acid doped and boron-reinforced PVA nanofibers (B1/BA1-PV (**a**), B1/BA1,5-PVA(c), B1/BA2-PVA(d), HR-SEM images of B1/BA1-PVA nanofibers (**b**) and EDS analysis of B1/BA2-PVA nanofiber mats

Using boric acid and elemental boron to combine hydrogen and boron atoms more effectively had a negative impact on fiber formation, as depicted in Fig. 8. As shown in Fig. 8a and b, the thickening effect of boric acid on the solution resulted in the formation of fibers with alternating large agglomerations of elemental boron particles distributed between the layers. When the boric acid content was increased to 1.5% by weight, a thin layer accumulated between the fibrous structures. As shown in Fig. 8c, these strata consisted of a solution that did not transform into fibers because its viscosity exceeded the optimal values. When the boric acid concentration in the solution was raised above 2%, the resulting viscous gel formed a continuous composite layer interspaced with very thin nanofibers (Fig. 8d). The EDS analysis depicted in Fig. 8e reveals that the composite layer's boron content reached 41 wt%, which is analogous to the composition's 10 wt% boron content. By fusing PVA with high concentrations of boric acid and elemental boron, the final composite layer was infused with the highest atomic percentage of boron. By combining boric acid and elemental boron, it is understood that borate ester layers envelop elemental boron particles during fiber structure assembly. As the concentration of boric acid increases, the borate ester layer increases further without harming the fiber layer. In nanocomposites produced from a solution containing 2% boric acid by weight, the fiber structure is known to be preserved, but the borate ester layer surrounding the fiber layer is not evident. This modification eliminates the porosity problem, which is considered a disadvantage of fiber for shielding applications, and creates a composite structure with greater strength than fiber.

### Radiation shielding performance of PVA nanofiber reinforced with boron and boric acid

The modulation of neutron, gamma, and X-ray attenuation characteristics was regulated by fabricating thin fibrous mats with diverse compositions, with an average thickness of 10 µm. Initially, fiber composite mats with dimensions of 10 × 10 cm^2^ were subjected to low-energy neutron irradiation, followed by three distinct detector measurements. The shielding percentages were calculated by computing the mean value of three distinct signals detected by the detector. Simultaneously, the assessment of gamma, x-ray, and neutron shielding was computed using Phy-X software [30]. The composite fiber densities and related compositions used in the Phy-X program are listed in Table 2.

**Table 2:** Neutron shielding test results

Sample Code	First reading (counts)	Second reading (counts)	Third reading (counts)	Average reading (counts)
Without Sample	1668	1653	1646	1653
Neat PVA	1573	1577	1569	1573
BA1-PVA	1533	1534	1534	1533
BA1,5-PVA	1519	1533	1520	1524
BA2-PVA	1510	1502	1509	1507
B0,5-PVA	1564	1550	1554	1556
B1-PVA	1528	1511	1526	1521
B2-PVA	1529	1544	1520	1531
B1/BA1-PVA	1477	1487	1486	1483
B1/BA1,5-PVA	1449	1462	1450	1453
B1/BA2-PVA	1435	1422	1430	1429

The process of reinforcement involves the incorporation of borate esters as well as boron nanoparticles into PVA molecular structure through modifications. The addition of boron atoms into PVA nanofibers has been found to enhance neutron shielding capabilities. The boron content in the nanofibers was systematically increased to achieve better shielding capacity. Table 2 displays the augmented average shielding percentages of PVA nanofibers, which were generated by incorporating boron using various starting materials into the solutions. The fast neutron removal cross-section values were analyzed through calculations conducted using the Phy-X program. Results revealed an inverse correlation between the shielding properties and the concentration of boron in the nanofibers, as shown in Table 3. It could be concluded that the incorporation of boric acid can result in an increase in the neutron shielding capacity up to approximately 9%.

**Table 3:** Neutron shielding percent and fast neutron removal croos section results

Sample Code	Neutron shielding percent (%)	FNRCS^*^ (1/cm)
Without sample	0	0
Neat PVA	4.84	0.116
BA1-PVA	7.26	0.130
BA1,5-PVA	7.81	0.126
BA2-PVA	8.84	0.122
B0,5-PVA	5.87	0.180
B1-PVA	7.99	0.201
B2-PVA	7.39	0.182
B1/BA1-PVA	10.29	0.205
B1/BA1,5-PVA	12.10	0.210
B1/BA2-PVA	13.56	0.213

^*^FNRCS fast neutron removal cross-section (1/cm)

The findings indicate that incorporating elemental boron into PVA nanofibers increased neutron shielding by approximately 8%, as observed through the average values measured. The increase in the weight percentage of elemental boron in the solution decreased the shielding percentage despite the rise in boron content. This can be attributed to the degradation of fiber morphology that occurred during the electrospinning process. It is known that when boric acid and elemental boron are used together and incorporated into the PVA polymer matrix, the neutron shielding values increase above 10 percent, and even with the B1/BA2-PVA sample, this value is 13.56 percent for a 10-micron-thick nanocomposite fiber mat. This increase was made possible by incorporating boron into the PVA polymer matrix, whose molecular chain structure was modified with boric acid, by distributing elemental boron uniformly throughout the matrix, and by minimizing the pore rate in the structure.

All theoretical calculations made with the Phy-X program were made based on the density and composition ratios found in Table 4.

**Table 4:** The compositions used in PhyX, including their description and component content

Sample Code	Density	Phy-X Composition
Neat PVA	1.2	**1**C4540H9080O2270
BA1-PVA	1.4244	**0.80**C4540H9080O2270 + **0.20**H3BO3
BA1,5-PVA	1.3998	**0.74**C4540H9080O2270 + **0.26**H3BO3
BA2-PVA	1.3912	**0.66**C4540H9080O2270 + **0.34**H3BO3
B0,5-PVA	1,9333	**0.90**C4540H9080O2270 + **0.10**B
B1-PVA	2,2569	**0.80**C4540H9080O2270 + **0.20**B
B2-PVA	2,1838	**0.66**C4540H9080O2270 + **0.34**B
B1/BA1-PVA	2,3901	**0.66**C4540H9080O2270 + **0.17**B + **0.17**H3BO3
B1/BA1,5-PVA	2,4611	**0.64**C4540H9080O2270 + **0.15**B + **0.21**H3BO3
B1/BA2-PVA	2.5371	**0.58**C4540H9080O2270 + **0.14**B + **0.28**H3BO3

Bold represents the proportions of materials in the composition. Their total for each composition must be 1 (100%)

**Fig. 9:**
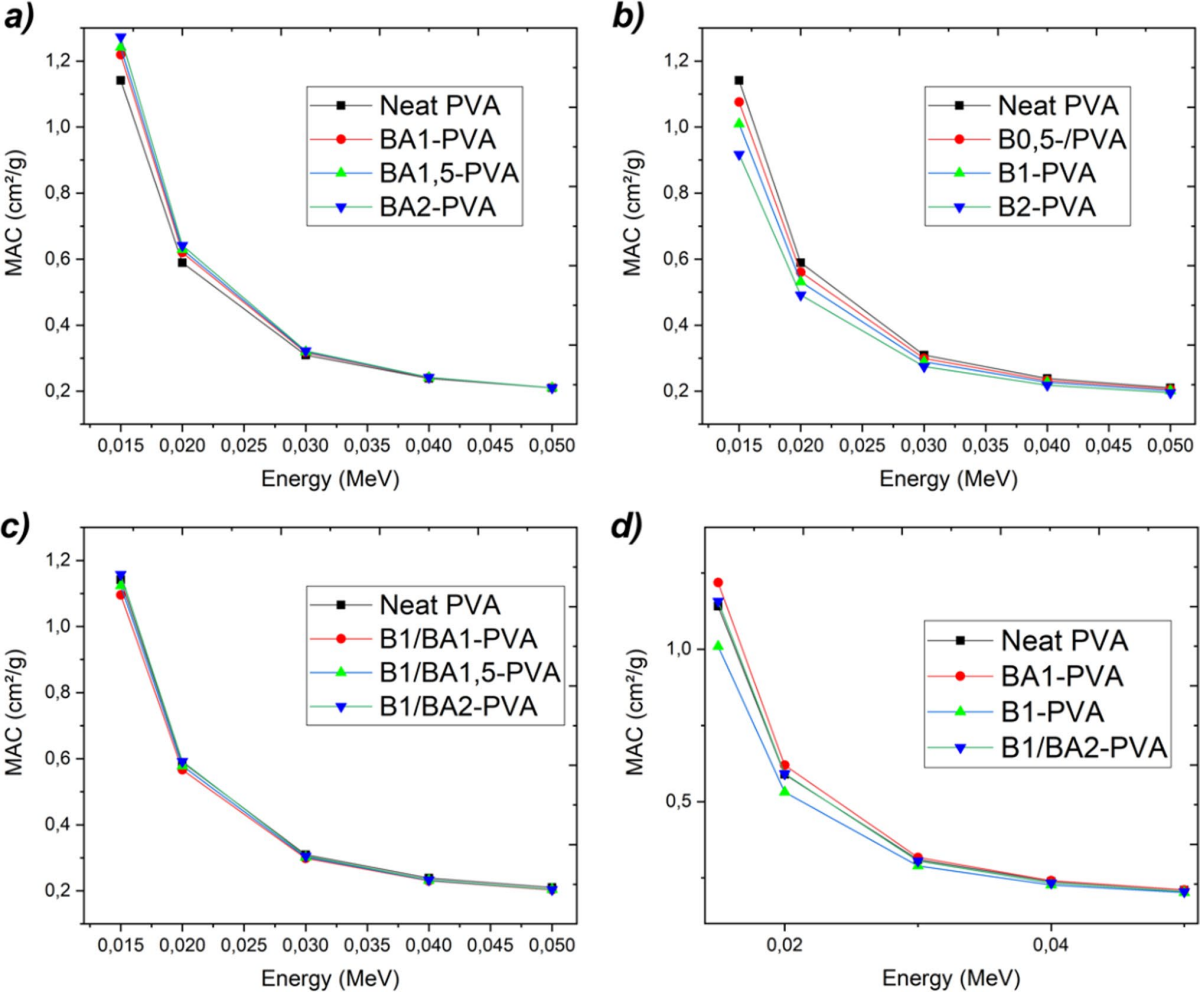
Variation of the photon energy versus mass attenuation coefficient according to the variation of the components in the compositions (boric acid doped PVA nanofibers (**a**), elemental boron reinforced PVA nanofibers (**b**), boric acid plus elemental boron reinforced PVA nanofibers (**c**) and comparison all (**d**) (obtained by Phy-X/PSD)

Figure 9 depicts the calculated mass attenuation coefficient variation range at low energies (0.015–0.05 MeV) for boric acid, elemental boron, and boric acid and elemental boron-supplemented PVA nanofibers, respectively. The observed correlation between the mass attenuation coefficient and adding boric acid can be attributed to hydrogen and boron atoms within the compound. In contrast, elemental boron supplementation decreased due to the decrease in hydrogen mass weight. This result may be significant, endorsing the importance of hydrogen in comprehending the mass attenuation coefficient as a variable absorption cross-section, where the effective area is defined per unit mass rather than per particle.

**Fig. 10:**
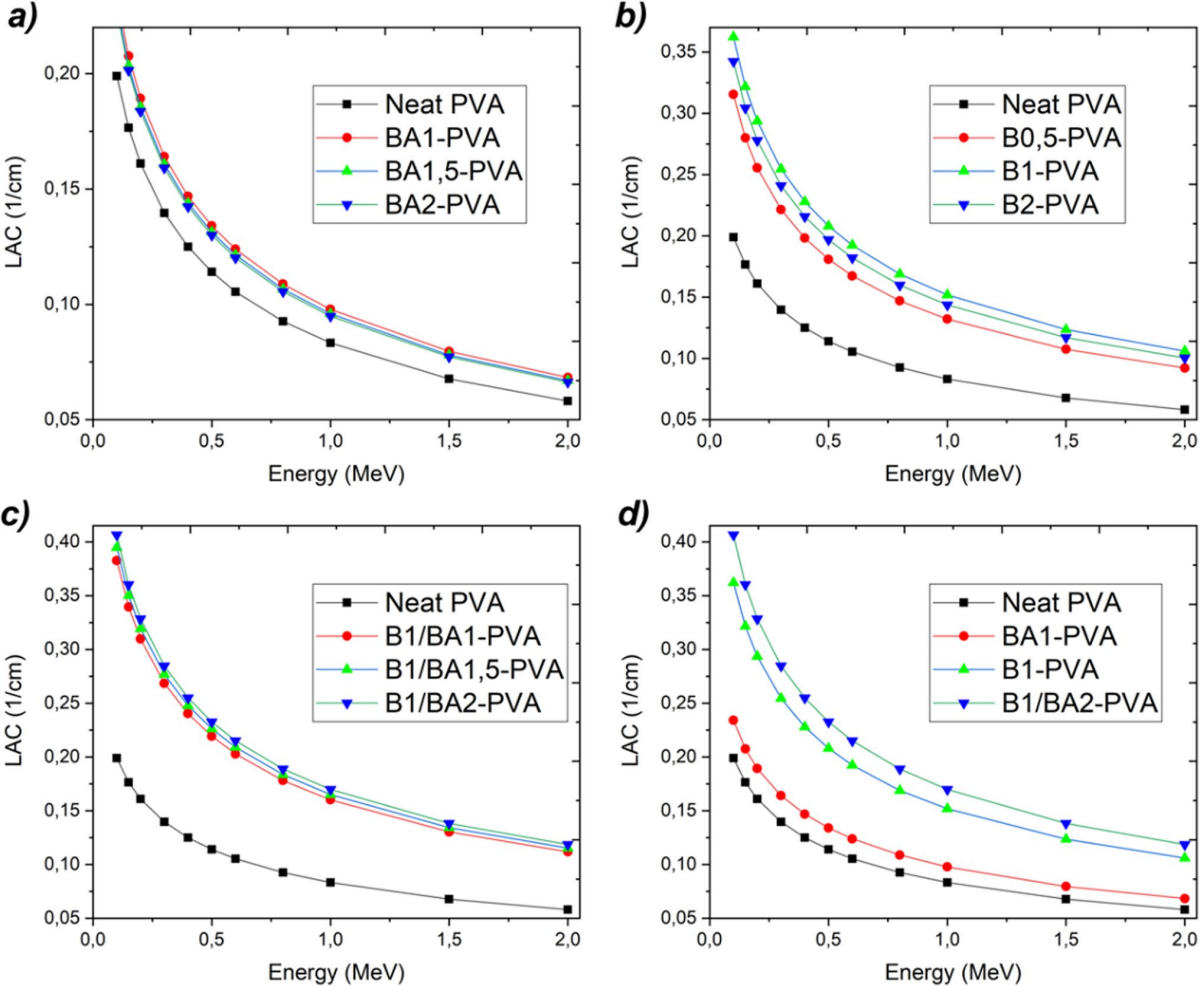
Variation of the photon energy versus linear attenuation coefficient according to the variation of the components in the compositions (boric acid doped PVA nanofibers (**a**), elemental boron reinforced PVA nanofibers (**b**), boric acid plus elemental boron reinforced PVA nanofibers (**c**) and comparison all (**d**) (obtained by Phy-X/PSD)

Figure 10 depicts the variation of the linear attenuation coefficient, a constant that characterizes the proportion of incident photons attenuated in a monoenergetic beam per unit thickness of a material, based on the composition of the material. The relationship between the linear attenuation coefficient and various parameters, including atomic number, the physical density of the absorber material, and density values, has been determined. It was observed that the linear attenuation coefficient was also increased as these factors were increased. Figure 10a depicts a decrease in the linear attenuation coefficients of PVA nanofibers, which can be attributed to the decrease in density from 1.42 to 1.39 g/ml caused by the addition of boric acid. Due to this justification, the linear attenuation coefficient was modified directly with a focus on preserving the integrity of the fiber structure and the homogenous distribution of materials. The decline of these two crucial factors resulted in a decrease in the linear attenuation coefficient, as a decrease in density would follow. The linear attenuation coefficient of PVA nanofibers reinforced with elemental boron is depicted in Fig. 10b. Similar to the behavior observed in PVA nanofibers doped with boric acid, the linear attenuation coefficient of PVA nanofibers doped with elemental boron was directly proportional to their density. The B1-PVA composite, which contained the most elemental boron reinforcement while preserving the fibers’ integrity, exhibited the highest linear attenuation coefficient. As predicted, the linear attenuation coefficients of the nanofibers derived from the compositions depicted in Fig. 10c containing both elemental boron and boric acid reinforcements increased in response to the increased density. Comparing various compositions, it was found that the B1/BA2-PVA composite had the maximum density and material reinforcement, resulting in the highest linear attenuation coefficient.

**Fig. 11:**
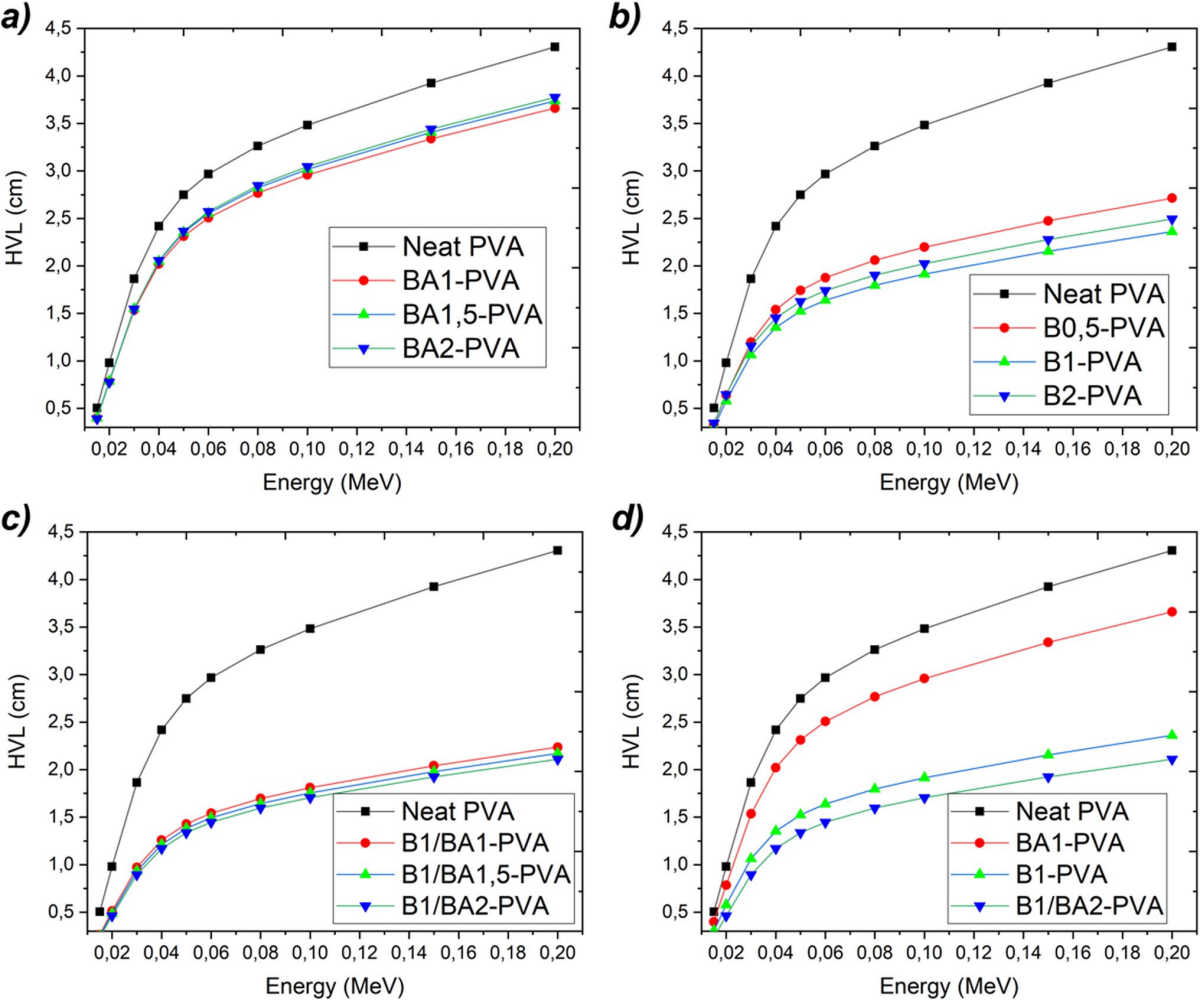
Variation of the photon energy versus half value layer according to the variation of the components in the compositions (boric acid doped PVA nanofibers (**a**), elemental boron reinforced PVA nanofibers (**b**), boric acid plus elemental boron reinforced PVA nanofibers (**c**) and comparison all (**d**) (obtained by Phy-X/PSD)

The half value layer, similar to the linear attenuation coefficient, indicates the capacity of X and gamma rays to penetrate a material. This property is influenced by the density and amount of material per unit area. The minimum amount of half value layer necessary to reduce the beam intensity emitted from the nanofiber source, composed of BA1-PVA with boric acid doping, as depicted in Fig. 11a, is sufficient to accomplish a fifty percent intensity reduction. The half value layer (HVL) is the material thickness required to reduce the beam intensity by half. As photon energy increases, so does the thickness of the material. Figure 11 graphs also depict the increase in energy and thickness for this reason. In samples containing boric acid-impregnated PVA nanofibers, the reduction in half-beam intensity shielding was approximately 3.5 cm.

Conversely, in the samples of PVA nanofibers reinforced with elemental boron, the HVL decreased to approximately 2.2 cm. Figure 11c demonstrates that the HVL depth in nanofibers generated from compositions containing both materials simultaneously was less than 2 cm. Consequently, radiation protection can be effectively achieved by utilizing samples with a thickness of less than 2 cm that have been prepared with nanofibers derived from the composition B1/BA2-PVA.

## Conclusion

Our study concludes with a comprehensive analysis of the effect of boric acid and elemental boron supplementation on the microstructure of polyvinyl alcohol (PVA) nanofibers and their radiation shielding capability. By changing the concentration of boric acid and elemental boron, the diameter, morphology, and thermal properties of PVA nanofibers can be successfully modified. This flexibility facilitates the production of composites with enhanced properties for use in radiation shielding. In our study, elemental boron reinforced borate ester nanofibers are embedded between borate ester layers with the optimal composition of 2% boric acid and 1% elemental boron by weight, ensuring the structural integrity of the fibers. In light of simulations performed with the PhyX program and actual experiments conducted at the nuclear research center, the development of a 10-micron-thick nanocomposite that shields neutrons by more than 13% stands out as a significant advancement. Beyond advanced radiation capabilities, the ability to control nanofiber dimensions and tailor the composite microstructure expands their applications to include lightweight and flexible radiation-protective apparel, efficient filtration systems, and advanced aerospace materials.

## Data Availability

Data is available on request due to privacy/ethical restrictions.
